# Predictors of Severe Obesity in Low-Income, Predominantly Hispanic/Latino Children: The Texas Childhood Obesity Research Demonstration Study

**DOI:** 10.5888/pcd14.170129

**Published:** 2017-12-28

**Authors:** Meliha Salahuddin, Adriana Pérez, Nalini Ranjit, Steven H. Kelder, Sarah E. Barlow, Stephen J. Pont, Nancy F. Butte, Deanna M. Hoelscher

**Affiliations:** 1 Michael & Susan Dell Center for Healthy Living, Austin, Texas; 2 The University of Texas Health Science Center at Houston School of Public Health in Austin, Austin, Texas; 3 Population Health, Office of Health Affairs, University of Texas System, Austin, Texas; 4 Texas Children’s Hospital, Baylor College of Medicine, Houston, Texas; 5 Texas Department of State Health Services, Office of Science and Population Health, Austin, Texas; 6 University of Texas at Austin Dell Medical School, Austin, Texas; 7 US Department of Agriculture/Agricultural Research Service Children’s Nutrition Research Center, Houston, Texas; 8 Department of Pediatrics, Baylor College of Medicine, Houston, Texas

## Abstract

**Introduction:**

The objective of this study was to identify predictors of severe obesity in a low-income, predominantly Hispanic/Latino sample of children in Texas.

**Methods:**

This cross-sectional analysis examined baseline data on 517 children from the secondary prevention component of the Texas Childhood Obesity Research Demonstration (TX CORD) study; data were collected from September 2012 through February 2014. Self-administered surveys were used to collect data from parents of children who were aged 2 to 12 years, had a body mass index (BMI) in the 85th percentile or higher, and resided in Austin, Texas, or Houston, Texas. Multivariable logistic regression models adjusted for sociodemographic covariates were used to examine associations of children’s early-life and maternal factors (large-for-gestational-age, exclusive breastfeeding for ≥4 months, maternal severe obesity [BMI ≥35.0 kg/m^2^]) and children’s behavioral factors (fruit and vegetable consumption, physical activity, screen time) with severe obesity (BMI ≥120% of 95th percentile), by age group (2–5 y, 6–8 y, and 9–12 y).

**Results:**

Across all ages, 184 (35.6%) children had severe obesity. Among children aged 9 to 12 years, large-for-gestational-age at birth (odds ratio [OR] = 2.31; 95% confidence interval [CI], 1.13–4.73) was significantly associated with severe obesity. Maternal severe obesity was significantly associated with severe obesity among children aged 2 to 5 years (OR = 2.67; 95% CI, 1.10–6.47) and 9 to 12 years (OR = 4.12; 95% CI, 1.84–9.23). No significant association was observed between behavioral factors and severe obesity in any age group.

**Conclusion:**

In this low-income, predominantly Hispanic/Latino sample of children, large-for-gestational-age and maternal severe obesity were risk factors for severe obesity among children in certain age groups. Promoting healthy lifestyle practices during preconception and prenatal periods could be an important intervention strategy for addressing childhood obesity.

## Introduction

Childhood obesity is a major public health challenge in the United States because of its high prevalence, adverse metabolic effects, racial/ethnic and socioeconomic disparities, and high economic costs. The prevalence of obesity among children leveled off between 2003–2004 and 2009–2010 in the United States (1); however, the prevalence of severe obesity increased (2). The National Health and Nutrition Examination Survey showed that the prevalence of severe obesity among children aged 2 to 19 years was 3.8% in 1999–2000 and 5.9% in 2011–2012 (3). The health and economic consequences of obesity (2,4,5) make it imperative to understand the associated risk factors. The determinants of severe obesity may be similar to the lifestyle, environmental, familial, and societal risk factors for overweight or obesity (4). Because obesity originates as early as the prenatal period (6), it is important to examine early-life, maternal, and childhood behavioral factors. Few studies have investigated the risk factors and protective factors of severe obesity. One study among a nationally representative sample of children in the United States identified early-life and maternal factors such as crossing the 85th percentile of BMI at an early age, maternal pre-pregnancy severe obesity, gestational diabetes, and Latino ethnicity as risk factors and certain behavioral factors, such as attending a child care facility and eating fruit at least weekly at kindergarten age, as protective factors for severe obesity (7). Thus, opportunities exist to explore and identify potentially modifiable factors for severe obesity among children.

The objective of this study was to assess the associations of early-life, maternal, and behavioral factors with severe obesity among a sample of low-income, predominantly Hispanic/Latino children aged 2 to 12 years with overweight, obesity, and severe obesity who participated in the secondary prevention randomized controlled trial (RCT) of the Texas Childhood Obesity Research Demonstration (TX CORD) study.

## Methods

The TX CORD study was an integrated, systems-oriented model that incorporated primary and secondary obesity prevention approaches across multiple sectors (primary health care clinics, early care and education centers, elementary schools, and community organizations) and levels (child, family, community, and environment/policy) in Austin, Texas, and Houston, Texas. The secondary prevention component was an RCT that compared an intensive 12-month childhood obesity management program with a primary care provider–based intervention in the primary catchment area. Baseline data for the secondary prevention study were collected from September 2012 through February 2014 (8,9). The target population resided in low-income medically underserved areas and consisted of children aged 2 to 12 years who were eligible for public health insurance. Catchment areas in Austin and Houston were determined by using an index that comprised data on income and racial/ethnic composition and data from geographical information systems. Details of the study design and methodology of selection and distribution of the catchment areas are provided elsewhere (8,9). 

The RCT was originally designed with a sample size of 576 to provide sufficient statistical power to determine the effect of the intervention on the primary outcome, body mass index (BMI) *z* score (8). The study recruited 549 participants. We restricted this analysis to children born from 23.0 to 41.0 weeks of gestation (n = 517). We conducted a secondary analysis of cross-sectional data on baseline measures, before randomization.

For the RCT, children aged 2 to 12 years with overweight and obesity were recruited from the primary health care clinics in TX CORD catchment areas and randomized to either the intervention group or comparison group by age group (2–5 y [n = 149], 6–8 y [n = 173], and 9–12 y [n = 195]). The age groups encompassed the age range in the program’s funding guidelines and thus clustered children with similar verbal and physical skills by age. During the RCT, data were collected on anthropometric, physiologic, and fitness measures of children; anthropometric measures of parents; and dietary, physical activity, and psychosocial health measures of parents and children (8). The institutional review board of the University of Texas Health Science Center at Houston Committee for Protection of Human Subjects approved this analysis (Clinical Trial nos. NCT02724943 and HSC-SPH-11–0513).

### Measures

**Outcome.** The outcome of interest was severe obesity status. Anthropometric data, including child’s height and weight, were obtained by trained research staff members using standard equipment (8). BMI was calculated as weight in kilograms divided by height in meters squared (kg/m^2^). The Centers for Disease Control and Prevention’s 2000 growth charts were used to express each child’s BMI as sex-specific and age-specific BMI percentile and as a percentage of sex-specific and age-specific BMI above the 95th percentile (10). Children with a BMI that was 120% or more above the 95th percentile of sex-specific and age-specific BMI (2,11) were categorized as having severe obesity. Children with a BMI in the 85th percentile or above to less than 120% of BMI above the 95th percentile were categorized as having overweight or obesity.

**Exposures.** Two sets of exposures were of interest: early-life and maternal factors (large-for-gestational-age, exclusive breastfeeding ≥4 months, and maternal severe obesity) and child’s behavioral factors (fruit and vegetable consumption, physical activity, and the presence of television in the room where the child sleeps).

Two questions were used to ask parents about birth weight and gestational age of their child: 1) “What was your child’s weight at birth?” (parents were given open spaces to input numbers in pounds and ounces), and 2) “At how many weeks was your child born?” (parents were given open spaces to input numbers in weeks). Gestational age ranged from 23.0 to 46.0 weeks. To be consistent with research in which growth curves were used to calculate large-for-gestational-age (12), we restricted the study sample to children born from 23.0 weeks to 41.0 weeks. Using birth weight and gestational age, we computed sex-specific large-for-gestational-age (birth weight >90th percentile), adequate-for-gestational-age (birth weight in the 10th–90th percentile), and small-for-gestational-age (birth weight <10th percentile) variables, which correspond with the new intrauterine growth curves created and validated for US neonatal intensive care units (12). These 3 categories were collapsed into 2 categories: large-for-gestational-age (birth weight > 90th percentile) and not large-for-gestational-age (birth weight ≤90th percentile).

Parents were asked 2 questions about infant breastfeeding practices: “For how long was your child breastfed?” and “How old was your child when infant formula milk was introduced?” The response options were “less than 1 week,” “less than 1 month,” “2–3 months,” “4–6 months,” and “more than 6 months” for the breastfeeding question, and “didn’t give formula,” “less than 1 month,” “2–3 months,” “4–6 months,” and “more than 6 months” for the formula-feeding question. Children who were breastfed for at least 4 months and not introduced to formula until 4 months were classified as exclusively breastfed for the first 4 months of life or more.

Maternal severe obesity at the time of study enrollment was defined as having a BMI of 35.0 kg/m^2^ or more; thus it included both class 2 and 3 obesity (13). Procedures for measuring height and weight of mothers were similar to procedures used for their children (8).

Child’s fruit and vegetable consumption was determined by parental report of how many times a child ate a fruit, ate a vegetable, or drank fruit juice “yesterday”; response options were “no,” “1 time,” “2 times,” and “3 or more times” for each item. We summed the responses for fruits, vegetables, and fruit juice when the parent selected 1 time or fewer for the fruit juice item. Otherwise, we summed the responses for fruit and vegetables only.

Child’s physical activity level was operationalized as whether the child met the current physical activity recommendations of 60 minutes per day of physical activity in the past 7 days. The item was measured by using the following question: “During the past 7 days, on how many days was your child physically active for a total of ≥60 minutes per day? (Add up all the time your child spent in any kind of physical activity that increased their heart rate and made them breathe hard some of the time.)”; response options were “0 days,” “1 day,” “2 days,” “3 days,” “4 days,” “5 days,” “6 days,” and “7 days.” Children were classified as meeting physical activity recommendations if parents indicated 7 days of activity.

Screen time was determined by parental report (yes or no) of a television in the room where the child sleeps.

**Covariates.** Covariates were selected on the basis of research and plausible direct or indirect association with the outcome variable (6,7,14–16): sex of child, parent-reported race/ethnicity of child, poverty-income ratio (PIR), parental marital status, and parent’s physical activity level. Race/ethnicity was categorized as Hispanic/Latino or non-Hispanic black. Seven (1.4%) children were neither Hispanic/Latino nor non-Hispanic black, and they were included in the non-Hispanic black category. PIR was based on household size and income and was calculated by using poverty guidelines of the US Department of Health and Human Services (17). PIR was classified as less than 125% or 125% or more. Parents’ physical activity was operationalized as whether or not they were physically active for 30 minutes per day on 5 or more days per week.

### Data analysis

The RCT was powered for the primary outcome by age group: 2 to 5 years, 6 to 8 years, and 9 to 12 years (8); thus, the secondary-data analyses were conducted by age group. We calculated descriptive statistics for the total sample, by age group, for the outcome of interest as medians and interquartile ranges for continuous variables and frequencies and percentages for categorical variables.

We used multiple logistic regression models separately for each age group to assess the associations of early-life and maternal factors with severe obesity, after adjusting for the covariates. We repeated the same modeling strategy to assess the associations of behavioral factors with severe obesity, after adjusting for the covariates. Approximately 10% of the data for the exposures and covariates were missing. Thus, we conducted a complete case analysis for the 2 analytic models (the early-life and maternal model [n = 461] and the behavioral model [n = 505]). We calculated odds ratios (ORs) and 95% confidence intervals (CIs). We performed a sensitivity analysis using an alternative approach of multiple imputation to account for missing data that were assumed to be missing at random (18). The imputation models (2 models for each age group) included the variables used in corresponding analytic models. Ten data sets were imputed, using chained equations, for each of the 6 imputation models. The data sets were then pooled for the final statistical analysis. A *P* value of < .05 was considered significant. Additionally, we compared sociodemographic characteristics of responders and nonresponders. STATA software version 14.0 (StataCorp LLC) was used for data analysis.

## Results

Across all ages, 184 (35.6%) children had severe obesity. Among participants aged 2 to 5 years, one-quarter (38 of 149) had severe obesity (Table 1). Among participants aged 6 to 8 years (69 of 173) and 9 to 12 years (77 of 195), about 40% had severe obesity. Overall, about half (263 of 517) of the participants were girls, most (449 of 517) were Hispanic/Latino, and most (434 of 517) had a PIR of less than 125%. Participants who did not complete questionnaires were more likely to be boys and have a PIR of 125% or more. 

**Table 1:** Baseline Characteristics of Participants, by Age Group, in the Secondary Prevention Randomized Controlled Trial of the Texas Childhood Obesity Research Demonstration Study, 2012–2014^a^

Characteristic	All	Overweight or Obesity^b^	Severe Obesity^c^
**Age group 2–5 y**			
No.	149	111	38
Female, n (%)	73 (49.0)	53 (47.8)	20 (52.6)
Hispanic/Latino, n (%)	133 (89.3)	99 (89.2)	34 (89.5)
Poverty-income ratio^d^ <125%, n (%)	128 (85.9)	98 (88.3)	30 (79.0)
Parent married, n (%)	112 (75.2)	82 (73.9)	30 (79.0)
Parent physically active ≥30 minutes for ≥5days/week, n (%)	25 (16.8)	14 (12.6)	11 (29.0)
Large for gestational age, n (%)	25 (16.8)	15 (13.5)	10 (26.3)
Exclusively breastfed for ≥4 months, n (%)	43 (28.9)	32 (28.8)	11 (29.0)
Maternal severe obesity (BMI ≥35 kg/m^2^), n (%)	49 (32.9)	31 (27.9)	18 (47.4)
Child’s fruit and vegetable consumption^e^, median (IQR)	3.0 (2.0-4.0)	3.0 (2.0-4.0)	2.5 (1.0-4.0)
Child physically active ≥60 minutes per day for 7 days/week, n (%)	37 (24.8)	28 (25.2)	9 (23.7)
Presence of television in the room where the child sleeps, n (%)	105 (70.5)	76 (68.5)	29 (76.3)
**Age group 6–8 y**			
No.	173	104	69
Female, n (%)	93 (53.8)	58 (55.8)	35 (50.7)
Hispanic/Latino, n (%)	145 (83.8)	93 (89.4)	52 (75.4)
Poverty-income ratio^d^ <125%, n (%)	142 (82.1)	86 (82.7)	56 (81.2)
Parent married, n (%)	127 (73.4)	80 (76.9)	47 (68.1)
Parent physically active ≥30 minutes for ≥5days/week, n (%)	23 (13.3)	16 (15.4)	7 (10.1)
Large for gestational age, n (%)	36 (20.8)	18 (17.3)	18 (26.1)
Exclusively breastfed for ≥4 months, n (%)	54 (31.2)	39 (37.5)	15 (21.7)
Maternal severe obesity (BMI ≥35 kg/m^2^), n (%)	50 (28.9)	24 (23.1)	26 (37.7)
Child’s fruit and vegetable consumption^e^, median (IQR)	2.0 (1.0-4.0)	2.0 (1.0-4.0)	3.0 (2.0-4.0)
Child physically active ≥60 minutes per day for 7 days/week, n (%)	27 (15.6)	15 (14.4)	12 (17.4)
Presence of television in the room where the child sleeps, n (%)	130 (75.1)	74 (71.2)	56 (81.2)
**Age group 9–12 y**			
No.	195	118	77
Female, n (%)	97 (49.7)	66 (55.9)	31 (40.3)
Hispanic/Latino, n (%)	171 (87.7)	106 (89.8)	65 (84.4)
Poverty-income ratio^d^ <125%, n (%)	164 (84.1)	97 (82.2)	67 (87.0)
Parent married, n (%)	147 (75.4)	88 (74.6)	59 (76.6)
Parent physically active ≥30 minutes for ≥5days/week, n (%)	27 (13.9)	18 (15.3)	9 (11.7)
Large for gestational age, n (%)	58 (29.7)	28 (23.7)	30 (39.0)
Exclusively breastfed for ≥4 months, n (%)	55 (28.2)	34 (28.8)	21 (27.3)
Maternal severe obesity (BMI ≥35 kg/m^2^), n (%)	55(28.2)	24 (20.3)	31 (40.3)
Child’s fruit and vegetable consumption^e^, median (IQR)	3.0 (1.0-4.0)	3.0 (1.0-4.0)	2.0 (2.0-4.0)
Child physically active ≥60 minutes per day for 7 days/week, n (%)	25 (12.8)	19 (16.1)	6 (7.8)
Presence of television in the room where the child sleeps, n (%)	130 (66.7)	78 (66.1)	52 (67.5)

Abbreviations: BMI, body mass index; IQR, interquartile range.

a Missing data for all variables of interest was approximately 10%; thus, these data were not included in the table.

b Overweight or obesity defined as BMI ≥85th percentile to <120% above the 95th percentile of the sex-specific and age-specific BMI.

c Severe obesity defined as BMI that was ≥120% above the 95th percentile of sex-specific and age-specific BMI.

d Poverty-income ratio was based on household size and income and was calculated by using poverty guidelines of the US Department of Health and Human Services poverty guidelines (17).

e Number of times child ate fruits and vegetables “yesterday.”

Large-for-gestational-age children were more likely to have severe obesity, but the relationship was significant only for children aged 9 to 12 years (OR = 2.31; 95% CI, 1.13–4.73) (Table 2). Maternal severe obesity was associated with severe obesity among children aged 2 to 5 years (OR = 2.67; 95% CI, 1.10–6.47) and 9 to 12 years (OR = 4.12; 95% CI, 1.84–9.23). Children aged 6 to 8 years whose mothers had severe obesity had 1.58 higher odds of having severe obesity, but this association was not significant (OR = 1.58; 95% CI, 0.74–3.35). Being breastfed exclusively for the first 4 months of life or more was not significantly associated with severe obesity in any of the age groups.

**Table 2:** Complete Case Analysis: Associations of Early-Life, Maternal, and Behavioral Factors With Severe Obesity, by Age Group, at Baseline, in Sample of Children in the Secondary Prevention Randomized Controlled Trial of the Texas Childhood Obesity Research Demonstration Study, 2012–2014

Factor	Adjusted Odds Ratio^a^ (95% Confidence Interval)	*P *Value
**Age Group 2–5 y**		
**Early-life and maternal factors (n = 137)^b^**		
Large for gestational age (birth weight >90th percentile)	2.36 (0.85–6.53)	.10
Exclusively breastfed for ≥4 months	1.69 (0.63–4.51)	.30
Maternal severe obesity (BMI ≥35 kg/m^2^)	2.67 (1.10–6.47)	.03
**Child’s behavioral factors (n = 147)^b^**		
Fruit and vegetable consumption (no. of times “yesterday”)	0.89 (0.70–1.13)	.33
Physically active ≥60 minutes per day for 7 days/week	0.76 (0.29–2.02)	.59
Presence of television in the room where the child sleeps	1.91 (0.72–5.05)	.19

**Age Group 6–8 y**		
**Early-life and maternal factors (n = 152)^b^**		
Large for gestational age (birth weight >90th percentile)	1.48 (0.62–3.53)	.38
Exclusively breastfed for ≥4 months	0.55 (0.26–1.20)	.13
Maternal severe obesity (BMI ≥35 kg/m^2^)	1.58 (0.74–3.35)	.24
**Child’s behavioral factors (n = 172)^b^**		
Fruit and vegetable consumption (no. of times “yesterday”)	1.11 (0.91–1.35)	.29
Physically active ≥60 minutes per day for 7 days/week	1.29 (0.53–3.14)	.57
Presence of television in the room where the child sleeps	1.76 (0.80–3.87)	.16

**Age Group 9–12 y**		
**Early-life and maternal factors (n = 172)^b^**		
Large for gestational age (birth weight >90th percentile)	2.31(1.13–4.73)	.02
Exclusively breastfed for ≥4 months	0.94 (0.43–2.03)	.87
Maternal severe obesity (BMI ≥35 kg/m^2^)	4.12 (1.84–9.23)	.001
**Child’s behavioral factors (n = 186)^b^**		
Fruit and vegetable consumption (no. of times “yesterday”)	0.98 (0.81–1.19)	.85
Physically active ≥60 minutes per day for 7 days/week	0.38 (0.13–1.10)	.08
Presence of television in the room where the child sleeps	1.09 (0.57–2.07)	.80

Abbreviation: BMI, body mass index.

a Adjusted for child’s sex, child’s race/ethnicity, poverty income ratio <125% or not, parental marital status, and parent’s physical activity (≥30 minutes per day of physical activity for ≥5 days/week).

b Sample sizes in categories differ from the sample sizes in Table 1 because of missing data.

None of the behavioral factors (children’s fruit and vegetable consumption, physical activity, or screen time) was significantly associated with severe obesity in our sample (Table 2), although the associations were in the expected direction for these factors. With every unit increase in fruit and vegetable consumption, children aged 2 to 5 years and 9 to 12 years had lower odds of severe obesity, but not significantly lower. Children aged 2 to 5 years and 9 to 12 years who met physical activity recommendations were at lower risk of severe obesity, but, again, not significantly. Similarly, having a television in the room where the child sleeps was more common among children with severe obesity in all age groups, but the relationship was not significant. The sensitivity analysis that used multiple imputation methods confirmed our findings (Table 3).

**Table 3:** Sensitivity Analysis Using Multiple Imputation: Associations of Early-Life, Maternal, and Behavioral Factors With Severe Obesity, by Age Group, at Baseline, in Sample of Children in the Secondary Prevention Randomized Controlled Trial of the Texas Childhood Obesity Research Demonstration Study, 2012–2014

Factor	Adjusted Odds Ratio^a^ (95% Confidence Interval)	*P* Value
**Age Group 2–5 y (n = 149)**		
**Early-life and maternal factors**		
Large for gestational age (birth weight >90th percentile)	1.95 (0.72–5.28)	.19
Exclusively breastfed for ≥4 months	1.35 (0.53–3.45)	.53
Maternal severe obesity (BMI ≥35 kg/m^2^)	2.90 (1.24–6.83)	.02
**Child’s behavioral factors**		
Fruit and vegetable consumption (no. of times “yesterday”)	0.87 (0.69–1.11)	.27
Physically active ≥60 minutes per day for 7 days/week	0.78 (0.30–2.03)	.60
Presence of television in the room where the child sleeps	1.92 (0.72–5.12)	.19

**Age Group 6–8 y (n = 173)**		
**Early-life and maternal factors**		
Large for gestational age (birth weight >90th percentile)	1.66 (0.74–3.70)	.22
Exclusively breastfed for ≥4 months	0.50 (0.24–1.04)	.06
Maternal severe obesity (BMI ≥35 kg/m^2^)	1.65 (0.72–3.78)	.24
**Child’s behavioral factors**		
Fruit and vegetable consumption (no. of times “yesterday”)	1.11 (0.92–1.35)	.28
Physically active ≥60 minutes per day for 7 days/week	1.29 (0.53–3.13)	.57
Presence of television in the room where the child sleeps	1.79 (0.82–3.93)	.15

**Age Group 9–12 y (n = 195)**		
**Early-life and maternal factors**		
Large for gestational age (birth weight >90th percentile)	2.13 (1.07–4.24)	.03
Exclusively breastfed for ≥4 months	1.06 (0.52–2.17)	.88
Maternal severe obesity (BMI ≥35 kg/m^2^)	4.58 (2.08–10.11)	<.001
**Child’s behavioral factors**		
Fruit and vegetable consumption (no. of times “yesterday”)	0.97 (0.80–1.17)	.77
Physically active ≥60 minutes per day for 7 days/week	0.39 (0.14–1.15)	.09
Presence of television in the room where the child sleeps	1.03 (0.55–1.94)	.93

Abbreviation: BMI, body mass index.

a Adjusted for child’s sex, child’s race/ethnicity, poverty income ratio <125% or not, parental marital status, and parent’s physical activity (≥30 minutes per day of physical activity for ≥5 days/week).

## Discussion

Our findings indicated that large-for-gestational-age and maternal severe obesity were significantly associated with severe obesity after adjusting for child’s sex, child’s race/ethnicity, PIR, parental marital status, and parent’s physical activity level among low-income, predominantly Hispanic/Latino children with overweight and obesity. However, in the same cohort, neither being breastfed exclusively for the first 4 months of life or more nor behavioral factors (children’s fruit and vegetable consumption, physical activity, and screen time) were significantly associated with severe obesity.

Studies have reported an association between large-for-gestational-age and childhood overweight/obesity (19,20), but none has examined severe obesity. A meta-analysis of 66 prospective studies worldwide reported that infants with high birth weight (>4,000 g) had 1.6 times greater odds of childhood overweight/obesity than infants with normal birth weight (2,500–4,000 g) (21). In line with our findings, one large prospective study conducted in The Netherlands (2001–2005) reported that maternal obesity was more likely to be associated with childhood obesity (OR = 5.02, 95% CI, 2.97–8.45) when compared to maternal normal weight (22). In our study, being breastfed exclusively for at least 4 months was a potentially (but not significantly) protective factor for severe obesity among children aged 6 to 8 years and 9 to 12 years. Evidence for the link between breastfeeding and childhood overweight and obesity is inconsistent, but one meta-analysis of 10 prospective studies reported that any breastfeeding, compared with formula feeding, reduced the risk of childhood overweight by 15% (6). Overall, our study adds to evidence that the prenatal environment influences the development of severe obesity and should be a target of intervention.

We observed no relationships between behavioral factors and severe obesity when we compared children with overweight and obesity to children with severe obesity. This finding may have been due to several reasons, including a similarity in lifestyle factors between children with overweight or obesity and children with severe obesity (4) or a similar reporting bias in the 2 groups in our study. Or, perhaps behavioral factors have little influence on the severity of weight gain when comparing children with overweight and obesity to those with severe obesity.

Our study has limitations, many of which are common to secondary analyses of cross-sectional data. The nonresponse rates in the exposures and covariates were around 10%, resulting in a reduced sample size in the complete case analysis. Our outcome measure was binary, which further limited the power of the study. We cannot make temporal inferences because the study was cross-sectional. Some degree of confounding bias, selection bias, reporting bias (including selective reporting of behavioral factors), or measurement error was likely. We could not control for important variables such as maternal cigarette smoking during pregnancy, gestational weight gain, gestational diabetes, adiposity rebound, and early-life dietary practices associated with childhood overweight/obesity (6,23,24). Research participants were children with overweight and obesity; thus, we cannot extrapolate our findings to normal-weight children. Furthermore, all participants consented to be part of the study, and their characteristics might differ from those of nonparticipants, thus subjecting the study to further selection bias. The outcome measure, severe obesity, measured as the percentage of sex-specific and age-specific BMI above the 95th percentile, does not reflect true body fat mass. Being breastfed exclusively was measured as being breastfed exclusively for 4 months or more, not for 6 months or more, which is recommended by the American Academy of Pediatrics (25); the response option in the TX CORD study indicated 4 months or more. Finally, the self-reported and retrospectively collected data were subject to measurement and recall bias; however, we would expect this bias to affect responses similarly in all categories.

Despite the limitations, our study has several strengths. Our study sample comprised predominantly Hispanic/Latino children from low-income families in Texas, and we used reliable and validated measures (12,26,27). Although a few studies have examined the determinants of severe obesity among children (7,28), none, to the best of our knowledge, has investigated the risk factors of severe obesity in an underrepresented and at-risk population. A recent review reported that the lifestyle, environmental, familial, and societal risk factors were similar for severe obesity and overweight/obesity among children (4). Our findings indicate that severe obesity has some of the same risk factors as obesity in Hispanic/Latino children.

More than one-third of the children in our study had severe obesity, a finding that is consistent with previous findings (29). Policy makers, health practitioners, researchers, and community health workers should work together to design culturally appropriate interventions and obesity-prevention strategies to stem the rising rate of severe obesity in low-income Hispanic/Latino populations, beginning as early as the preconception period. These strategies should target multiple modifiable factors, incorporate family-based lifestyle modifications (30), and be tailored toward population needs. Future studies should explore the associations of multiple early-life risk factors (maternal cigarette smoking during pregnancy, gestational weight gain, gestational diabetes, adiposity rebound, and early-life dietary practices) with childhood severe obesity and the mechanisms linking them.

Our study suggests that large-for-gestational-age and maternal severe obesity contribute to severe obesity in a low-income, predominantly Hispanic/Latino sample of children. Furthermore, our study indicates that several determinants of severe obesity, such as dietary behaviors, physical activity, and screen time are not different from the determinants of overweight and obesity in this population.

## References

[1] Flegal KM , Carroll MD , Kit BK , Ogden CL . Prevalence of obesity and trends in the distribution of body mass index among US adults, 1999–2010. JAMA 2012;307(5):491–7. 10.1001/jama.2012.39 22253363

[2] Kelly AS , Barlow SE , Rao G , Inge TH , Hayman LL , Steinberger J , Severe obesity in children and adolescents: identification, associated health risks, and treatment approaches: a scientific statement from the American Heart Association. Circulation 2013;128(15):1689–712. 10.1161/CIR.0b013e3182a5cfb3 24016455

[3] Skinner AC , Skelton JA . Prevalence and trends in obesity and severe obesity among children in the United States, 1999–2012. JAMA Pediatr 2014;168(6):561–6. 10.1001/jamapediatrics.2014.21 24710576

[4] Bass R , Eneli I . Severe childhood obesity: an under-recognised and growing health problem. Postgrad Med J 2015;91(1081):639–45. 10.1136/postgradmedj-2014-133033 26338983

[5] Finkelstein EA , Khavjou OA , Thompson H , Trogdon JG , Pan L , Sherry B , Obesity and severe obesity forecasts through 2030. Am J Prev Med 2012;42(6):563–70. 10.1016/j.amepre.2011.10.026 22608371

[6] Weng SF , Redsell SA , Swift JA , Yang M , Glazebrook CP . Systematic review and meta-analyses of risk factors for childhood overweight identifiable during infancy. Arch Dis Child 2012;97(12):1019–26. 10.1136/archdischild-2012-302263 23109090 PMC3512440

[7] Flores G , Lin H . Factors predicting severe childhood obesity in kindergarteners. Int J Obes 2013;37(1):31–9. 10.1038/ijo.2012.168 23147114

[8] Hoelscher DM , Butte NF , Barlow S , Vandewater EA , Sharma SV , Huang T , Incorporating primary and secondary prevention approaches to address childhood obesity prevention and treatment in a low-income, ethnically diverse population: study design and demographic data from the Texas Childhood Obesity Research Demonstration (TX CORD) study. Child Obes 2015;11(1):71–91. 10.1089/chi.2014.0084 25555188 PMC4696423

[9] Oluyomi AO , Byars A , Byrd-Williams C , Sharma SV , Durand C , Hoelscher DM , The utility of Geographical Information Systems (GIS) in systems-oriented obesity intervention projects: the selection of comparable study sites for a quasi-experimental intervention design — TX CORD. Child Obes 2015;11(1):58–70. 10.1089/chi.2014.0054 25587670 PMC4696445

[10] Kuczmarski R , Ogden C , Guo S . 2000 CDC growth charts for the United States: methods and development. Vital and Health Statistics, series 11, no. 246. Hyattsville (MD): US Department of Health and Human Services, Centers for Disease Control and Prevention, National Center for Health Statistics; 2002. 12043359

[11] Flegal KM , Wei R , Ogden CL , Freedman DS , Johnson CL , Curtin LR . Characterizing extreme values of body mass index-for-age by using the 2000 Centers for Disease Control and Prevention growth charts. Am J Clin Nutr 2009;90(5):1314–20. 10.3945/ajcn.2009.28335 19776142

[12] Olsen IE , Groveman SA , Lawson ML , Clark RH , Zemel BS . New intrauterine growth curves based on United States data. Pediatrics 2010;125(2):e214–24. 10.1542/peds.2009-0913 20100760

[13] Flegal KM , Kit BK , Orpana H , Graubard BI . Association of all-cause mortality with overweight and obesity using standard body mass index categories: a systematic review and meta-analysis. JAMA 2013;309(1):71–82. 10.1001/jama.2012.113905 23280227 PMC4855514

[14] Stettler N , Zemel BS , Kumanyika S , Stallings VA . Infant weight gain and childhood overweight status in a multicenter, cohort study. Pediatrics 2002;109(2):194–9. 10.1542/peds.109.2.194 11826195

[15] Wang L , Mamudu HM , Wu T . The impact of maternal prenatal smoking on the development of childhood overweight in school-aged children. Pediatr Obes 2013;8(3):178–88. 10.1111/j.2047-6310.2012.00103.x 23042596

[16] Shields L , O’Callaghan M , Williams GM , Najman JM , Bor W . Breastfeeding and obesity at 14 years: a cohort study. J Paediatr Child Health 2006;42(5):289–96. 10.1111/j.1440-1754.2006.00864.x 16712560

[17] US Department of Health and Human Services. 2011–2012 Data documentation, codebook, and frequencies: demographic variables and sample weights (DEMO_G). In: National Health and Nutrition Examination Survey. 2013. https://wwwn.cdc.gov/nchs/nhanes/2011-2012/DEMO_G.htm#INDFMPIR. Accessed November 7, 2017.

[18] Enders CK . Analyzing longitudinal data with missing values. Rehabil Psychol 2011;56(4):267–88. 10.1037/a0025579 21967118

[19] Chiavaroli V , Derraik JGB , Hofman PL , Cutfield WS . Born large for gestational age: bigger is not always better. J Pediatr 2016;170:307–11. 10.1016/j.jpeds.2015.11.043 26707580

[20] Xie C , Wang Y , Li X , Wen X . Childhood growth trajectories of etiological subgroups of large for gestational age newborns. J Pediatr 2016;170:60–6.e1, 5. 10.1016/j.jpeds.2015.11.031 26687713

[21] Schellong K , Schulz S , Harder T , Plagemann A . Birth weight and long-term overweight risk: systematic review and a meta-analysis including 643,902 persons from 66 studies and 26 countries globally. PLoS One 2012;7(10):e47776. 10.1371/journal.pone.0047776 23082214 PMC3474767

[22] Gaillard R , Durmuş B , Hofman A , Mackenbach JP , Steegers EAP , Jaddoe VWV . Risk factors and outcomes of maternal obesity and excessive weight gain during pregnancy. Obesity (Silver Spring) 2013;21(5):1046–55. 10.1002/oby.20088 23784909

[23] Robinson SM , Marriott LD , Crozier SR , Harvey NC , Gale CR , Inskip HM , Variations in infant feeding practice are associated with body composition in childhood: a prospective cohort study. J Clin Endocrinol Metab 2009;94(8):2799–805. 10.1210/jc.2009-0030 19435826

[24] Rolland-Cachera MF , Deheeger M , Maillot M , Bellisle F . Early adiposity rebound: causes and consequences for obesity in children and adults. Int J Obes 2006;30(Suppl 4):S11–7. 10.1038/sj.ijo.0803514 17133230

[25] The American Academy of Pediatrics. AAP reaffirms breastfeeding guidelines. 2012. https://www.aap.org/en-us/about-the-aap/aap-press-room/pages/aap-reaffirms-breastfeeding-guidelines.aspx. Accessed July 18, 2017.

[26] Tomeo CA , Rich-Edwards JW , Michels KB , Berkey CS , Hunter DJ , Frazier AL , Reproducibility and validity of maternal recall of pregnancy-related events. Epidemiology 1999;10(6):774–7. 10.1097/00001648-199911000-00022 10535796

[27] Hoelscher DM , Day RS , Kelder SH , Ward JL . Reproducibility and validity of the secondary level School-Based Nutrition Monitoring student questionnaire. J Am Diet Assoc 2003;103(2):186–94. 10.1053/jada.2003.50031 12589324

[28] Gittner LS , Ludington-Hoe SM , Haller HS . Infant obesity and severe obesity growth patterns in the first two years of life. Matern Child Health J 2014;18(3):613–24. 10.1007/s10995-013-1285-y 23775247

[29] Lo JC , Chandra M , Sinaiko A , Daniels SR , Prineas RJ , Maring B , Severe obesity in children: prevalence, persistence and relation to hypertension. Int J Pediatr Endocrinol 2014;2014(1):3. 10.1186/1687-9856-2014-3 24580759 PMC3976673

[30] Hoelscher DM , Kirk S , Ritchie L , Cunningham-Sabo L ; Academy Positions Committee. Position of the Academy of Nutrition and Dietetics: interventions for the prevention and treatment of pediatric overweight and obesity. J Acad Nutr Diet 2013;113(10):1375–94. 10.1016/j.jand.2013.08.004 24054714

